# Adapting Citizen Science to Improve Health in an Occupational Setting: Preliminary Results of a Qualitative Study

**DOI:** 10.3390/ijerph17144917

**Published:** 2020-07-08

**Authors:** Mandy van den Berge, Gerben Hulsegge, Henk F. van der Molen, Karin I. Proper, H. Roeline W. Pasman, Lea den Broeder, Sietske J. Tamminga, Carel T. J. Hulshof, Allard J. van der Beek

**Affiliations:** 1 Department of Public and Occupational Health, Amsterdam Public Health Research Institute, Amsterdam UMC, Vrije Universiteit Amsterdam, 1081 BT Amsterdam, The Netherlands; gerben.hulsegge@tno.nl (G.H.); karin.proper@rivm.nl (K.I.P.); hrw.pasman@amsterdamumc.nl (H.R.W.P.); 2 Sustainable Productivity and Employability, The Netherlands Organization for Applied Scientific Research, TNO, 2316 ZL Leiden, The Netherlands; 3 Department of Public and Occupational Health, Amsterdam Public Health Research Institute, Coronel Institute of Occupational Health, Netherlands Center for Occupational Diseases, Amsterdam UMC, University of Amsterdam, 1100 DD Amsterdam, The Netherlands; h.f.vandermolen@amsterdamumc.nl (H.F.v.d.M.); s.tamminga1@amsterdamumc.nl (S.J.T.); c.t.hulshof@amsterdamumc.nl (C.T.J.H.); 4 Centre for Nutrition, Prevention and Health Services, National Institute for Public Health and the Environment, 3721 MA Bilthoven, The Netherlands; lea.den.broeder@rivm.nl; 5 Faculty of Health, Amsterdam University of Applied Sciences, 1105 BD Amsterdam, The Netherlands

**Keywords:** blue-collar workers, worksite health promotion, unhealthy lifestyle, citizen science

## Abstract

Health interventions often do not reach blue-collar workers. Citizen science engages target groups in the design and execution of health interventions, but has not yet been applied in an occupational setting. This preliminary study determines barriers and facilitators and feasible elements for citizen science to improve the health of blue-collar workers. The study was conducted in a terminal and construction company by performing semi-structured interviews and focus groups with employees, company management and experts. Interviews and focus groups were analyzed using thematic content analysis and the elements were pilot tested. Workers considered work pressure, work location and several personal factors as barriers for citizen science at the worksite, and (lack of) social support and (negative) social culture both as barriers and facilitators. Citizen science to improve health at the worksite may include three elements: (1) knowledge and skills, (2) social support and social culture, and (3) awareness about lifestyle behaviors. Strategies to implement these elements may be company specific. This study provides relevant indications on feasible elements and strategies for citizen science to improve health at the worksite. Further studies on the feasibility of citizen science in other settings, including a larger and more heterogeneous sample of blue-collar workers, are necessary.

## 1. Introduction

Blue-collar workers have on average a poorer health than white-collar workers and are often less educated [1,2,3,4,5,6]. One of the main factors explaining the poor health of blue-collar workers is unhealthy lifestyle. Blue-collar workers smoke more often, have less healthy diets, and have lower levels of leisure-time physical activity than white-collar workers [2,7,8]. Another important determinant of health is their work environment [7,8,9,10,11]. For example, blue-collar workers more often have jobs with physically demanding work and lower job autonomy than white-collar workers [2,12]. Both unfavorable work characteristics and unhealthy lifestyle factors are associated with an increased risk for health problems, such as musculoskeletal disorders, cardiovascular diseases, and type 2 diabetes [1,2,7,13]. Thus, the promotion of health in blue-collar workers is urgently needed.

The worksite is a potential setting to promote health among workers, including blue-collar workers [14,15]. However, Worksite Health Promotion Programs (WHPPs) often do not reach blue-collar workers and even if they participate they seem to drop out earlier and not comply as well as white-collar workers [15,16,17]. One of the reasons for the limited reach and participation of blue-collar workers in WHPPs is that many WHPPs are predominantly cognition-based, which probably does not fit the needs, skills, and capacities of more practically-oriented blue-collar workers [3,6]. For example, Tonnon et al. [18] conducted a qualitative study on barriers and facilitators for implementation of a lifestyle intervention to reduce cardiovascular disease risk among construction workers. They concluded that employees did not have the knowledge and skills to correctly estimate their own health risk, which was a barrier to participate in a lifestyle intervention [18]. In addition, another qualitative study regarding the successful recruitment of adults with a low socioeconomic position (SEP) into community-based lifestyle programs [19] acknowledged the educational differences of researchers and target groups as a barrier. Therefore, the experts highly recommend consultation of the target groups in the design of promotion strategies for a successful recruitment [19]. A recent review on the effectiveness of WHPPs [20] showed that WHPPs were more effective among population groups such as blue-collar workers if the participants were involved in the design and implementation of WHPPs. By involving the target population more actively during the design stage of a study, WHPPs can be tailored to the possibilities and needs of the targeted population and of their worksites, and therefore might also improve reach and compliance [20].

Active involvement of the target population is possible by using participatory research methods [21,22,23]. Citizen science is such participatory method, which holds promise to improve health by designing and testing better tailored WHPPs and thereby improving reach, compliance, and also effectiveness. Citizen science was first used in the natural sciences as a way to facilitate data collection [24,25] but has been deployed in other fields as well, including public health [26,27,28,29,30,31]. Citizen science is defined as “the general public engagement in scientific research activities when citizens actively contribute to science either with their intellectual effort or surrounding knowledge or with their tools and resources” [32]. Hinckson et al. [33] described that citizen science provides a way to facilitate partnerships between researchers and citizens. Researchers share scientific knowledge with citizens regarding health issues in their community [19,33]. There are different types of involvement of citizens to participate in citizen science. The two most common strategies are described as contributory and co-created [34]. Contributory citizen science includes a top-down structure with limited engagement of citizens in the scientific research process and uses a common citizen science model. Co-created citizen science applies the engagement of citizens in most or all steps of research by using a bottom-up approach [34,35,36]. The goals of co-creation are commonly focused on including empowerment and direct action focused on shared problems.

An example of co-created, bottom-up citizen science approach is the study of den Broeder et al. [29], conducted in disadvantaged neighborhoods in the Netherlands, where residents were invited and trained to become citizen scientists. The citizen scientists were actively engaged in research in order to collect, report, and analyze data regarding the influence of their neighborhood on their health [29]. The study entailed the target community identifying ways to actively engage in improving the health of their community. Other studies have applied citizen science in broader communities to improve health by changing the environment [26,30,31]. However, little is known about the feasibility of citizen science in an occupational setting with predominantly blue-collar workers. Co-creation based citizen science may also be valuable in an occupational setting to actively engage blue-collar workers as citizen scientists and to improve health among blue-collar workers. Nonetheless, contextual factors largely differ between neighborhood and occupational settings [37]. For example changing worksites and long commutes after exhausting workdays do not apply in a neighborhood setting. Such differences ask for a different approach. For citizen science to be feasible in an occupational setting, more insight is needed in the needs and possibilities of blue-collar workers and the organizational setting.

Therefore, this preliminary study aimed to adapt citizen science to improve health in an occupational setting. This is, to our knowledge, the first study that investigates the barriers, facilitators and feasible elements for citizen science in an occupational setting. Two study objectives were defined: (1) to determine the barriers and facilitators for citizen science to improve health in an occupational setting with predominantly blue-collar workers, and (2) to identify which elements of citizen science would be feasible to apply in an occupational setting to improve health among predominantly blue-collar workers.

## 2. Materials and Methods

### 2.1. Study Design

This qualitative study used different qualitative techniques, including semi-structured interviews, focus groups, expert meetings, and small pilot tests. To collect data for the future effect- and process evaluation, participants filled out a questionnaire before the start of the pilot test. This questionnaire was based on questions used in previous studies and validated questionnaires [38,39,40,41,42,43,44,45]. To provide an insight in the characteristics of the participated employees in interviews and focus groups, information on age, job function, and physical workload was presented in the current study.

### 2.2. Participants and Recruitment

The study population included blue-collar workers between 18 and 67 years of age. These workers were recruited from companies in the construction sector and the transport and logistics sector, because both of these sectors employ a high proportion of blue-collar workers. The companies were selected by searching the internet and by using the network of the researchers. The companies were approached by e-mail to invite their participation in this study. Subsequently, after a few weeks, the management of the companies were called and eventually visited, in order to explain the study in more detail. In total, ten companies in the construction sector and one in the transport and logistics sector were contacted. In the end, two large sized companies (between 300 and 650 employees) decided to participate.

The participating construction company is involved in building, renovation, restoration, and maintenance services. In total, the company has 309 employees, out of whom 30 blue-collar are workers from the (internal) carpentry factory and 103 are blue-collar workers with various job functions, mainly working at temporary construction sites. Furthermore, the company has 176 white-collar workers, including office workers, supervisors, middle and higher management.

A large container terminal company from the transport and logistics sector also participated in the study. The company offers shipping and landside services, including transport, storage, and maintenance services for containers, gate and reefer services. The participating terminal has 615 employees, 445 of whom are blue-collar workers and 170 of whom are white-collar workers.

The employees in both companies were predominantly male. Maximum variation sampling method was used to select participants based on ages, job functions, and perspectives towards lifestyle and health. Due to the fact that both companies intended to implement the intervention company-wide, a number of white-collar workers was also included. The sampling was carried out by Human Resource (HR) advisors or prevention workers at the companies, who invited the employees to participate in the study.

The HR advisors or prevention workers of the companies were asked to invite other important stakeholders (mostly from the management teams) to participate in the development and implementation of the citizen science study. No further motivation strategies, such as rewards, were used to increase participation.

### 2.3. Medical Ethics

The Medical Ethical Committee of VU University Medical Center (Amsterdam, the Netherlands) declared that it was not necessary to seek ethical approval to conduct this study and had no objection to the study being carried out (reference number: 2018.138). All participants signed informed consent forms prior to their participation in (various) parts of this study.

### 2.4. Data Collection

**Figure 1:**
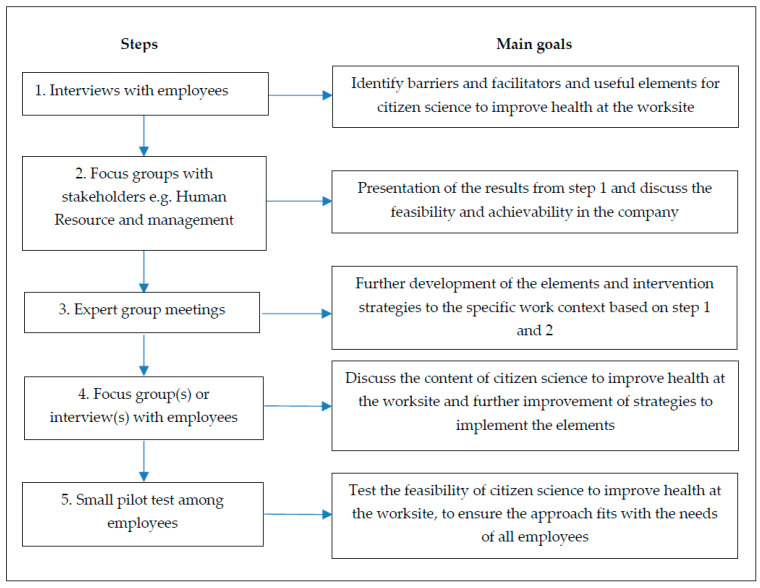
A flowchart of the different steps taken to adapt citizen science to improve health in an occupational setting.

All data were collected during an iterative process using interviews and focus groups at the worksite during working hours. To adapt citizen science to improve health in an occupational setting the following inductive steps were taken to collect the data (Figure 1).

#### 2.4.1. Step 1. Interviews with Employees

In the first phase of the study, semi-structured individual interviews with employees were conducted by the first author (M.v.d.B.). The main goal of the interviews was to identify barriers, facilitators (objective 1), and feasible elements of citizen science (objective 2) to improve health in an occupational setting. The interviews were guided by an interview topic list. The duration of the interviews ranged from 30 to 50 min. The interviews were audio recorded and transcribed verbatim. In addition, the researcher spent part of the day at the worksite together with the interviewed employees, to gain a sense of trust with the interviewees and more understanding and insight into their work tasks (by filling in a checklist, self-administered by the researcher). The interview guide and checklists were generated by the researchers and based on topics from a previous citizen science study among low socioeconomic adults [29].

#### 2.4.2. Step 2. Focus Groups with Stakeholders

The main results of the interviews conducted in step 1 were discussed with the managers, prevention team, and HR advisors in a focus group interview in each company. During this meeting, citizen science elements named in step 1 were discussed and further adapted to the possibilities of the worksite and the organization. The duration of each of the focus groups was one and a half hours. The focus groups were audio recorded and transcribed verbatim. Two authors (M.v.d.B. and G.H.) carried out these focus groups. During the focus groups, the first researcher presented the findings derived from the interviews, asked questions, and led the discussions. The second researcher took notes and asked additional questions.

#### 2.4.3. Step 3. Expert Group Meetings

Based on the results of the interviews and focus groups (steps 1 and 2), a group of experts further adapted citizen science elements to the specific worksite settings and made materials (e.g., information brochures, diaries, fact sheets) and workshops suitable for blue-collar workers. The group consisted of experts in citizen science, experts from the national occupational health institute of the construction industry, experts/trainers in the communication of health information to people with low health literacy, and experienced researchers in occupational health.

#### 2.4.4. Step 4. Focus Groups with Employees

The interviewees from step 1 were contacted by e-mail to participate in focus groups, in order to discuss the content of citizen science and results of the focus groups with stakeholders (step 2) and to further improve the materials made by the expert group (step 3). At two locations of the construction company, focus groups were not possible, as only one or two employees were present at these locations. In these cases (*n* = 2) an interview was conducted instead of a focus group. In total, one focus group (*n* = 9) was conducted at the terminal company, and two focus groups (*n* = 4 and *n* = 5) and two individual interviews were conducted at the construction company. The duration of the focus group meetings and interviews ranged from 30-90 min. Due to the rotating shift schedule in the terminal company and the changing temporary construction sites in the construction company, it was not possible to include all those interviewees who participated in step 1 in focus groups as well. Instead, other employees (*n* = 5) (predominantly blue-collar workers) were asked to participate.

#### 2.4.5. Step 5. Small Pilot-Tests

The adapted citizen science approach to improve health was pilot-tested among a portion of the interviewees in both companies, as well as among two employees at each company who were not involved in the interviews or focus groups. The citizen science elements were tested to ensure that they were feasible and reflected the needs of all employees. The researchers performed the first interactive meeting, as intended in citizen science. After the pilot tests, employees were asked to give oral feedback regarding the materials so far. Employees also tested the personal diary for several days and were contacted by phone or e-mail to give their feedback.

### 2.5. Analysis

Interview and focus group transcripts were coded based on thematic content analysis using Atlas.ti software. To get familiar with the data, the first author (M.v.d.B.) read the data in-depth. After this, two authors (G.H. and M.v.d.B.) independently developed codes in five interviews to ensure reliability in the coding procedure. These coded interviews were discussed, and the codes were organized and categorized in broader themes. Based on these themes and codes, the first author (M.v.d.B.) coded the other interviews. The codes were discussed, and decisions were taken together (M.v.d.B. and G.H.). To ensure reliability, the codes were checked again by the second author (G.H.). These codes and themes were further extended if needed, and used to specify the feasible elements and strategies in steps 3–5. The self-administered checklist (step 1) was summarized and used by the researchers to gain more in-depth understanding of the data derived from the interviews and focus groups. These codes and themes were used to structure the results (Appendix A). Quotations were included to clarify themes. The interviews were held in Dutch. The quotations were translated by a native speaker. To check the translation to English, a cross check was performed by a second native speaker.

## 3. Results

### 3.1. Study Population

**Figure 2:**
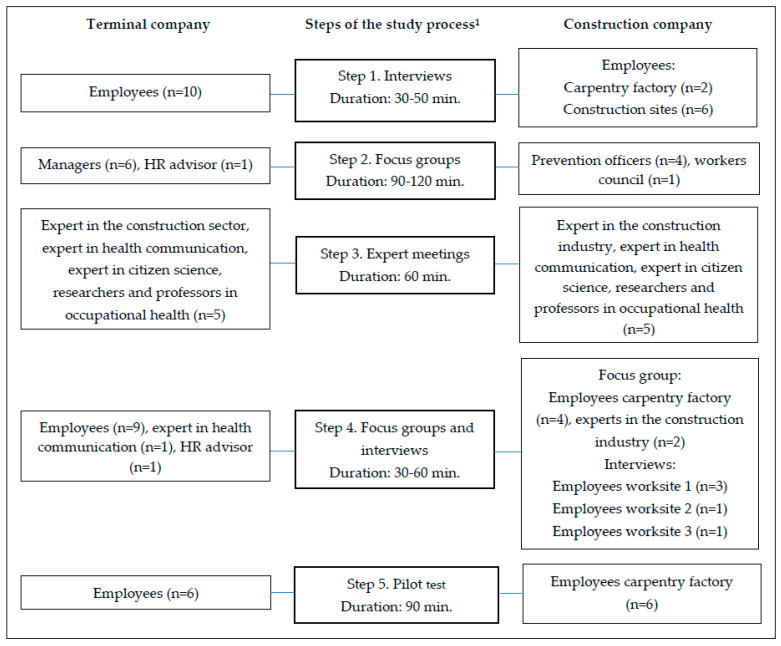
An overview of the participants in all steps of research. ^1^ See Figure 1 for an explanation of the steps. HR = Human Resources.

Figure 2 shows an overview of the participants for all steps of research in both companies. In total, 26 employees (19 blue-collar, 7 white-collar) participated in steps 1, 4 and 5 of data collection (construction company *n* = 12, terminal company *n* = 14). Most employees were men (96%). Sixteen employees filled out the questionnaire (*n* = 16). The ages of these employees ranged from 22 to 59 years. Of these employees, six employees rated their physical workload as heavy, five as intermediate, and five as low.

Due to the iterative process, the results are not described according to the steps of research that were followed, but described as barriers and facilitators and feasible elements for citizen science. Employees identified factors that might serve as barriers or serve to facilitate the use of citizen science aimed to improve health at the worksite (Table 1). The employees named several contextual and personal factors, which are further explained below. In general, employees considered most factors to be barriers and some factors were considered both barriers and facilitators. Based on these factors, feasible elements for citizen science to improve health at the worksite were further identified and designed during the steps that followed (Table 2). An overview of the codes and themes can be found in Appendix A.

### 3.2. Barriers and Facilitating Factors for Citizen Science to Improve Health at the Worksite

#### 3.2.1. Contextual Factors

First, employees working at the construction company’s temporary worksites perceived their work location as a factor that would inhibit the use of citizen science to improve health at the worksite. Due to their work at temporary construction sites, they often had to adapt to new circumstances, changing rules, and colleagues. In addition, the construction workers at temporary worksites had to deal with long, fatiguing commutes after exhausting workdays. They believed that these contextual factors might lead to difficulties for employing citizen science to improve health at their worksites, such as difficulties organizing meetings and effectively recruiting enough citizen scientists. These barriers did not apply to the employees working at the carpentry factory of the construction company because they work at a stationary worksite. At the terminal company, work location was seen as a barrier by employees, because the terminal was located in a remote industrial area and therefore many employees had to commute long distances. The long commutes reduced employees’ willingness to engage in other activities, such as participating in citizen science.

Carpenter at temporary worksites, age unknown:

*“As I see it, and speaking from personal experience, construction generally means having to work long hours. The work day starts at 06:45 and often includes a long commute, meaning you have to leave your house at the crack of dawn. The journey back home takes even longer due to traffic jams and the like – it’s exhausting doing that time and time again.”*

Second, both at the construction and the terminal company, work pressure was mentioned as a barrier for implementing citizen science to improve health at the worksite. Employees working at temporary construction sites experience high work pressure and an associated lack of time for other activities during working hours. This means that it would be rather difficult to actively take on the role of a citizen scientist. In contrast, employees working at the carpentry factory described their work as well distributed over time and, therefore, did not experience high work pressure, which might be a facilitator for employing citizen science.

At the terminal company, work tasks and pressures may change quickly due to factors such as delayed or changed transport schedules which sometimes lead to unexpectedly high work pressure. Another factor at the terminal company was the shift schedule, which was mentioned as both a possible barrier and a possible facilitator. Varied shift schedules might make it hard to recruit enough citizen scientists at the worksite and to organize meetings during or outside working hours. However, employees also suggested that the rotating schedule could improve participation in citizen science because after one group of employees has left a shift, others continue to work. According to them, this reduces work pressure and overtime duties, which may be helpful organizing citizen science activities after working hours.

Marine planner at the terminal company, between 35–49 years of age:

*“Yes. The workload is heavy, and if you want to work in this industry you need to be able to handle that. There are definitely periods that are incredibly busy. Some projects give you enough time to do everything and allow you to properly plan the work on a ship. But more often than not, you’re in a situation where only half of your plan is complete while the ship is already just around the corner. So you know you only have an hour before the work starts. In other words, go, go, go!”*

Third, most employees in both companies felt that the ‘masculine culture’ among colleagues discouraged talking about personal topics, such as lifestyle and health, and also discouraged participating as citizen scientists at the worksite. This was also underlined by the management of both companies. Therefore, social support and social culture at the worksite were considered as barriers for using citizen science to improve health at the worksite.

Focus group, management construction company:

*“Well I guess a lot of the guys here have this mentality of ‘I’m not a wuss, just keep powering on’. But if pressed, they’ll admit that they have a backache or some other ailment.”*

When employees were asked about social support from colleagues, the employees working at temporary construction sites mentioned that they had to deal with frequently changing colleagues, and, therefore, they regarded themselves more as individuals than as being part of a team. They thought this might be a barrier for using citizen science to improve health at the worksite, since colleagues might not listen to each other or even know each other at all.

Carpenter at temporary worksites, between 50–64 years of age:

*“Because we always work on big projects. And don’t forget, you guys [researchers] see each other every day – we don’t. For example, I might be working on a project with my colleagues, let’s say, and then be transferred to this project for four months and then to another project after that. This means I might not see my colleagues for 10 years because we are part of such a large organization.”*

In contrast, some employees working at the carpentry factory of the construction company and the terminal company felt as being part of a team and thought this was a facilitating factor for citizen science to be able to improve health at the worksite. Despite the ‘masculine culture,’ they felt that they could talk about a lot of things with colleagues and they thought colleagues advising each other might be more effective than an outsider telling them what to do. A few employees at the terminal company thought social support could be a facilitator to motivate colleagues with negative attitudes towards lifestyle and health.

Mechanic and safety trainer at the terminal company, between 35–49 years of age:

*“And sometimes we had some very difficult colleagues, who were very inflexible and would say ‘I’m not doing such and such’... I think the key, in such a situation, is to use the group to get these people to cooperate. If you have a group of people and a few enthusiastic people those are your sponsors use them to motivate others.”*

Likewise, some employees from the terminal company that participated were interested in playing the role of citizen scientist, by collecting data from interviews with colleagues for instance, but were also interested in promoting health and serving as motivators.

Regarding social support from the employers, most employees in both companies mentioned feeling positively about the work-related possibilities of their employer. They also felt their employer was involved personally with the employees, which they considered to be a facilitator for the use of citizen science to improve health at the worksite. Still, particularly at temporary construction sites, employees thought their employer should spend more time at the worksite listening to employees in order to have a better understanding of the daily issues at the worksite and to make clear agreements about how best to work together with the employees. They considered the absence of the employer to be a barrier to implementing citizen science because they felt that the employer had little insight into the experiences of employees at the worksite and did not feel appreciated by their employer. Therefore, without the employer spending time at the worksite, the employees were not highly motivated to actively participate in citizen science.

Focus group management, construction company:

*“Of course, one of the things people say is ‘we never, or hardly ever, see the executives’. This is a difficult dilemma for everyone – including the executives themselves. I don’t think it’s because it’s not important to them – but rather they lack the time and space for visits. These types of things become more difficult and complicated as an organization grows and even more so when you consider that we have several different locations. It just becomes very tricky.”*

#### 3.2.2. Personal Factors

In both companies, most employees were uncertain about whether their colleagues would be interested and open to talk about personal issues, such as lifestyle and health. This was mentioned as a reason why they would not join citizen science initiatives to address their health more actively as citizen scientist. As an explanation for this uncertainty, they suggested a lack of awareness and/or lack of/incorrect risk perceptions regarding health, lack of openness to changing their health behaviors, and negative attitudes.

Firstly, they believed that a lot of their colleagues actually had a lack of awareness and/or lack of/incorrect risk perceptions regarding health. As a consequence, they may not be motivated to change their lifestyles or participate actively in WHPPs in general.

Service mechanic at the terminal company, between 18–34 years of age:

*“Food and those kinds of things. It’s just easy - especially if it’s not a priority... I think lots of people don’t realize how unhealthy they are until they stop.”*

Secondly, some of the interviewees thought that their colleagues had negative attitudes and were not open to changes. These thoughts were confirmed by the management during focus groups. For example, if new tools at the worksite were offered by the employer, many employees resisted at first and it would take time before they could see the benefits of these new tools.

Machine worker and carpenter at the carpentry factory, between 18–34 years of age:

*“Yes I have... quite a lot of inflexible colleagues, who are not open to change. I mean, this is a great organization that takes care of everything. But a lot of people find it hard to adapt – they find change difficult.”*

The employees in both companies thought that, in general, their colleagues would have negative attitudes regarding health and might not be open to changing their behavior or talking about personal topics. They assumed that talking about health would be strange and they did not want to be different from the rest of the group. Therefore, they thought these negative attitudes would make it difficult to achieve results with citizen science to improve health at the worksite among employees.

### 3.3. Feasible Elements for Citizen Science to Improve Health at the Worksite

Besides the identified barriers and facilitators, codes and themes for citizen science elements were identified during the interviews and focus groups (step 1 and 2). During step 3–5, these codes and themes were extended if needed and used to specify the content and strategies for the citizen science elements. In the design of the elements, it was further elaborated on how to take these barriers and facilitators into account. After pilot testing, three main elements for citizen science to improve health at the worksite were identified, namely (1) improving knowledge and skills to improve lifestyle and health, (2) improving social support and social culture, and (3) creating awareness of lifestyle behaviors. An overview of the elements of citizen science to improve health at the worksite and the targeted barriers and facilitators can be found in Table 2.

#### 3.3.1. Improve Knowledge and Skills

In both companies, the employees considered trainings to increase knowledge and skills to be a key element for citizen science to improve health at the worksite. They believed that, some of their colleagues had negative attitudes towards health and might not be aware of their health risks because they believed they were already living healthy lives. Therefore, these employees thought that simple, short, and practical knowledge on lifestyle and health should be provided, to make workers more aware of their own lifestyles, which in turn would improve both their risk perception and their attitude towards lifestyle and health. This knowledge was provided via an interactive quiz designed with management, experts, and employees during steps 2–4. The quiz was pilot-tested and considered helpful by employees (step 5).

Furthermore, employees had thoughts on how to convey information towards their colleagues. First, narrative storytelling was considered to be a beneficial method because it could make health information more personal, which could in turn lead to a better understanding and acceptance of the message.

Machine worker and carpenter at the carpentry factory, between 18–34 years of age:

*“You sometimes see those videos about people breathing in dust and then getting serious illnesses so that they can’t live to see their children grow up. That is... well, I had just become a father and then it hits you hard. It makes you stop and think and makes you aware of the risks involved. I think that’s a good thing, but some people think it’s rubbish. But that’s mainly because a lot of people in this industry tend to be quite nonchalant. I think it’s better to be informed of the facts as they are.”*

Second, the information should not be given once, but repetition of the information was considered crucial to starting behavioral change. Third, personal goal setting was mentioned, because goals need to be personalized in order to see the relevance and get motivated.

#### 3.3.2. Social Support and Social Culture

Most interviewees thought it would not be feasible for employees to fulfill the role of citizen scientist at the worksite due to lack of time, lack of social support, and unsupportive social culture. Although the employees at the terminal company had these thoughts as well, some employees showed their interest in taking on the role of citizen scientist, but only if they would receive additional help. In addition, instead of this individual role as a citizen scientist, interviewees considered it essential to improve social support and create a more open social culture at the worksite to improve health. Therefore, social support and social culture were considered to be elements needed for citizen science to be able to improve health at the worksite. Interviewees mentioned that an interactive training to improve knowledge and skills should be given in small groups to create a safe setting and provide the possibility for employees to talk with one another about their experiences and perceived health problems. Personal stories from peers were perceived as useful and interviewees believed that employees would be more open to receiving help or advice from their own colleagues than from an outsider who is not familiar with their work at the construction site. This way, social support from colleagues and the social culture in general might be improved, which in turn might also lead to more openness to talking about health.

Focus group employees, terminal company:

*“Talking to others makes you realize they are facing the same issues we are. That can happen when talking to an internal staff member or when talking to someone outside the organization but also when you talk to someone... to two internal staff members or to two people from the outside. Everyone has different sleep habits, some people sleep really well and others have a lot of trouble sleeping. I am a good sleeper so I cannot tell someone who is having trouble sleeping what they should do to sleep better.”*

Besides social support from employees, employees working at temporary construction sites in particular emphasized that it would be beneficial if their employer would show them more appreciation and would attend the worksite more often to listen to the experiences and issues of the employees. The company management was aware of this problem. Therefore, it would be beneficial if the employer would attend group meetings at all temporary construction sites. At the terminal company, it was also mentioned that the employer should pay more attention to the employees’ health, for example by improving the food quality at the company restaurant.

#### 3.3.3. Creating Awareness of Lifestyle Behaviors

In order to make the knowledge and skills fit with the needs of the employees, interviewees in both companies often addressed awareness about their own health as an important element in changing behavior and increasing personal risk perceptions towards regarding health. Most employees thought more insight into their own health and health-related behavior would be needed to improve their motivation to change their lifestyle. Some employees explained that if they do not notice the problem, they will not consider improving their lifestyle actively.

Execution supervisor at the terminal company, age unknown:

*“Yes, being confronted with the hard facts is an option—telling someone about the horrible diseases they are at risk of developing. But is that really going to help? You don’t really know. It’s probably more helpful for someone to realize that they are not taking good care of themselves or to recognize their unhealthy behaviors, and then feel they would like to do something about that.”*

In both companies, interviewees believed gaining more insight into their own lifestyles, e.g., by collecting personal lifestyle data, might be a promising way to increase awareness. Although they expressed that this was challenging, the employees came up with various ideas. In the construction company, most of the employees thought keeping a diary would not work well with construction workers. They came up with various other ideas such as health checks, but also suggested taking pictures from physical exercise activities or groceries at the supermarket to gain more insight and awareness of their own lifestyles. In contrast, most employees as well as the management from the terminal company thought keeping a personal diary would be useful if the diary was tailored to the contextual work factors of the company. Therefore, the employees of the terminal company designed a personal diary with the help of the researchers, which was tested in step 5.

Manager health, safety, security and environment at the terminal company, age unknown:

*“I think it is much more effective to write down ‘I ate three frikandels (deep-fried sausages) today’ than to keep track of the calories you consumed in an app. I really believe that. Doing that for four weeks straight will give you a real wake-up call.”*

## 4. Discussion

This study exploring the possibility of citizen science to improve health at the worksite revealed several contextual and personal factors, mainly considered to be barriers to its use. These barriers included: Work pressure and work location (e.g., temporary construction sites among construction workers), lack of awareness, a lack of/incorrect risk perception, negative attitudes regarding health and a lack of openness towards lifestyle and health. In addition, social support and social culture were mentioned in both companies as possible barriers as well as facilitators. Furthermore, employees from the terminal company considered shift work as both a barrier and facilitator. Taking these barriers and facilitating factors into account, three main elements for applying citizen science to improve health at the worksites were identified, namely: improving knowledge and skills regarding lifestyle and health, improving social support and social culture, and creating awareness about current lifestyle behavior. The elements for citizen science to improve health at the workplace were comparable in both companies, but the strategies to implement these elements differed based on the perceived barriers, facilitators and possibilities.

The findings of present study are in line with other participatory approaches implemented at the worksites of blue-collar workers. For example, Lingard et al. [22] used participatory action research for health promotion among construction workers. They concluded that blue-collar construction workers are often interested in their health but felt restricted in their ability to change their behavior due to a lack of knowledge and work environmental factors such as the masculine culture. In addition, other studies identified social culture and the influence of masculinity at the worksite among blue-collar workers to have a negative impact on health behavior and their response on WHPP strategies, which underlines the need for changes in social support and social culture to improve health at the worksite [22,46].

Citizen science to improve health at the worksite was adapted by taking the needs, perceived possibilities, and perspectives of the target group into account. As a result, the present citizen science approach differs from previous studies which have conducted citizen science. For instance, den Broeder et al. [29] trained residents to become citizen scientists in order to collect data about the health of their neighbors [29]. In the current study, discussions were held with employees as to whether they would be interested in playing the role of citizen scientist. Most employees believed this would be challenging due to barriers such as lack of time, social culture, and personal factors (e.g., negative attitude towards health). This means that a total ‘citizen science approach’ or a very high level of co-creation [24,25] of the target population might not be feasible at the worksite of blue-collar workers. It was determined that it would, however, still be possible to include less intensive forms of citizen science or other participatory approaches by focusing on the three elements found in the current study. These insights from employees underline the importance of involving the target group in the design and implementation of WHPPs in order to fit their needs and possibilities. A recent systematic review on the effectiveness of WHPPs [20] also highly recommended the use of participatory approaches such as citizen science for WHPPs among blue-collar workers, since this leads to WHPPs that better fit the needs of the target group [20]. Still, the question remains open as to whether the citizen science approach in current study can still be classified as ‘citizen science’.

The first citizen science element considered as feasible in the current study was improving knowledge and skills regarding lifestyle and health. This is consistent with a review by Michie et al. [47], which described that providing information, facilitating goal setting, and prompting barrier identification are the most common techniques for changing behavior [47]. In addition, their review described that these techniques might work additively. For example, providing information about the benefits of behavior change might motivate people to change, while goal setting, identifying barriers, and social support might help to turn their motivation into action.

Secondly, improving social support and social culture was identified as element for citizen science to improve health at the worksite. However, Verdonk et al. [48] suggested that an individual program might also be important to attract men in programs that do not want a feeling of competition. Still, a review of Malik et al. [49] regarding the improvement of physical activity and underlined that a team-based approach works better than an individual approach [49]. A similar point was made in a qualitative study by Tonnon et al. [18] regarding perceived barriers and facilitators to the implementation of a lifestyle intervention in the Dutch construction industry. They pointed out that social support and social culture in the construction sector discouraged addressing problems about health [18]. The findings of these studies indicate the importance of improving social support and social culture by providing group-based, interactive meetings as part of citizen science aimed at improving health at the worksite.

The third element of citizen science to improve health in worksites was to create awareness of lifestyle behaviors. In line with the findings in our study, Tonnon et al. [18] suggested that employees did not feel at risk because they considered their lifestyle to be healthy and they did not have the knowledge and skills to correctly estimate their own health risk. Therefore, the combination of improving knowledge and skills and creating awareness of lifestyle behaviors are elements of citizen science that could be relevant to improve health at the worksite.

A systematic review of interventions that included an Assessment of Health Risks with Feedback [50] concluded that feedback on personal health information (e.g., by health screenings) may be a feasible strategy for creating awareness and increasing risk perception about personal lifestyle behavior [50]. Since we were unable to perform or use personal health screenings in this study and we wanted to actively involve the employees during the process of data collection, collecting personal data about lifestyle and health was included in order to raise awareness and increase employees’ perception of risks related to their own lifestyle behavior. The methods on how to collect personal lifestyle data were designed together with the employees and management of the companies (including personal diaries or pictures on lifestyle behaviors). These methods illustrate that other feasible approaches to raising awareness could be found which actively promote the participation of employees in data collection.

The preliminary results of this study contributes to literature on the feasibility of citizen science to improve health in occupational settings and, more specifically, at worksites of blue-collar workers. One strength was that this was achieved by using a step-by-step inductive process, meaning that each step was established on information derived from the previous steps. Furthermore, all data were collected at the worksite and, during all of five steps, the study population was actively involved as much as possible. This provided an in-depth needs assessment to adapt citizen science at the worksite.

However, this study also had some limitations. First, the sample of employees included in this study was small and most employees had a scarcity of time to actively participate in the study next to their regular work tasks. This lack of time among employees resulted in less participation and involvement of the target group and, therefore, might have led to more guidance from the researchers than initially planned. To make it somewhat less time intensive for employees and employer, all data were collected at the worksites and during working hours. Furthermore, in order to collect enough data and to avoid missing perspectives from employees in the adaptation of citizen science at the worksite, maximum variation sampling was used to select a heterogeneous group of predominantly blue-collar workers by age, job function and lifestyle and health. Still, the lack of time and small sample included in this study might have resulted in a less heterogeneous group of workers. For example, workers who were interested in lifestyle and health were more motivated to participate, or workers who experienced less work pressure in their job function. Therefore, this study provides preliminary findings regarding feasible elements for a citizen science approach to improve health in an occupational setting and was able to include a group of employees that was willing to participate. Second, since the companies were focused on an organization-wide implementation of citizen science to improve health, including not only blue-collar but also white-collar workers, it was decided to also include a group of white-collar workers in the study. As part of the citizen science approach, we followed this preference of the managers despite the study was initially predominantly focused on blue-collar workers. The inclusion of white-collar workers did not provide new elements, but extended and further elaborated on the relevant insights from blue-collar workers. Third, this study included two large sized companies from different sectors, which lacks the specific insights of small and medium size companies. Future research is needed to confirm the results from this preliminary study in a larger and more heterogeneous sample including employees from small and medium size companies.

Nevertheless, the current study provides first insights into citizen science’s ability to improve health at the worksite for blue-collar workers in different occupational settings. The current preliminary results show that the same elements of citizen science seems to be feasible in both companies, but that some intervention strategies differed because the citizen science approach was tailor-made and took barriers, facilitators, needs and possibilities of the organizational setting into account. An assessment of the organizational setting is an important first step for effective tailor-made WHPPs. Based on previous literature [18,22,47,49,50], we believe that the citizen science elements identified here might be feasible to other companies, but that the strategies to implement these elements may differ across companies. This is mainly because of the importance of assessing the organizational setting and adapting strategies used in citizen science to improve health at the worksite [51,52]. Therefore, further research should be conducted to determine to what extent citizen science needs to be adapted to other occupational settings.

## 5. Conclusions

This study is among the first studies that provides an insight into the adaptation of citizen science to improve health in an occupational setting and more specifically for blue-collar workers. To answer the first study objective, many contextual barriers and facilitators were identified for the use of citizen science to improve health at the worksite. In both companies, employees considered work pressure and work location as barriers, and social support and social culture to be both barriers and facilitators. Employees from the terminal company considered shift work to be both a barrier and a facilitator. Several personal factors were named to be barriers for citizen science to improve health, namely a lack of/incorrect risk perception, a lack of awareness and a negative attitude towards health, and a lack of openness to change their own behavior. To answer the second study objective, citizen science to improve health in an occupational setting may include three main elements: (1) improving knowledge and skills, (2) improving social support and social culture, and (3) creating awareness of lifestyle behavior. The strategies to implement the elements are slightly different at some points due to company specific barriers, facilitators, and possibilities. The identified elements and strategies need be considered and further elaborated within companies when designing and implementing WHPPs among blue-collar workers in other settings. This study was conducted in two large sized companies in two sectors and cannot, therefore, be generalized to other companies and sectors. Despite this, this study provides relevant indications for a feasible citizen science approach to improve health in an occupational setting. In addition, the small sample of employees and the scarcity of time among employees led to less participation and involvement of the target group than desirable. As a consequence, it remains open for debate whether the approach in the current study can still be called ‘citizen science’. Further studies on the feasibility of citizen science in other settings, including a larger and more heterogeneous sample of blue-collar workers, are necessary.

## Figures and Tables

**Table 1:** Barriers and facilitators for a citizen science approach to improve health at the worksite.

Category	Factors	Construction Company	Terminal Company
Context	Work location	Barrier	Barrier
	Work pressure	Barrier	Barrier
	Shift work	Not applicable	Barrier/Facilitator
	(Lack of) social support and (negative) social culture	Barrier/Facilitator	Barrier/Facilitator
Personal	Lack of openness for change	Barrier	Barrier
	Lack of/incorrect risk perception	Barrier	Barrier
	Negative attitude	Barrier	Barrier
	Lack of awareness	Barrier	Barrier

**Table 2:** Identified citizen science elements, strategies and targeted barriers and facilitators for citizen science to improve health in each company.

Citizen Science Element	Targeted Barriers/Facilitators	Strategies to Implement the Element	Included in
Improve knowledge and skills to improve lifestyle and health	Lack of/incorrect risk perception, lack of openness for change, lack of awareness, negative attitude	Content focused on lifestyle factors, mainly physical activity and dietary behavior, during shift work	T ^1^
		Content focused on physical activity during work and leisure time and dietary behavior in a physically demanding job	C 2
		Information provided on employee specific factsheets	C
		Information provided in a booklet	T
	Work location, work pressure	Short, repetitive meetings during working hours	C/T
	Lack of/incorrect risk perception, lack of openness for change, lack of awareness, negative attitude, work location, work pressure	Recurrent reminders of health messages via screens at the worksite	C
		Recurrent and reminder of health messages via e-mail or SMS	T
	(Lack of) social support and negative social culture	Interactive meetings	C/T
	Lack of/incorrect risk perception, lack of awareness, negative attitude, lack of openness for change	Narrative storytelling (a short video of an employee telling his personal story)	C
	Lack of/incorrect risk perception, lack of openness for change, lack of awareness	Personal goal setting	C/T
	(Lack of) social support and negative social culture, lack of awareness, negative attitude	Performing exercises during meetings	C
Improve social support and social culture	(Lack of) social support employees and negative social culture	Interactive, small group meetings with employees	C/T
	(Lack of) social support from the employer	Meetings facilitated by the prevention team and management of the company	C
		Providing a step counter	T
	(Lack of) social support, negative attitude, lack of openness for change, lack of awareness	Collect and discuss ideas on how to improve health at the worksite	C/T
		Active role in the decision-making process	C/T
	(Lack of) social support employees	Additional training of citizen scientists	T
Create awareness of lifestyle behavior	Lack of awareness, lack of/incorrect risk perception, negative attitude	Collect personal data on lifestyles (photos/videos)	C
		Collect personal data on lifestyle by keeping a diary and a step counter	T

^1^ Construction company. ^2^ Terminal company.

## Appendix A

**Table A1:** Codes and Themes.

Themes	Codes	Sub Codes
Barriers for citizen science	- Work environment	- Different worksites/work location - Working hours - Work pressure - Shift work - Physical workload - Other
	- Social support and social culture	- Employer (Appreciation) - Colleagues/subcontractors - Masculine culture - Different worksites
	- Financial resources	- Employer - Employee
	- Private life	
	- Personal/behavioral factors	- Age - Distrust - Patronized feeling - Shame - Not open for change - Negative attitude - No interest in lifestyle and health - Lack of awareness - Lack of/incorrect risk perception
Facilitators for citizen science	- Work environment	- Shift work
	- Social support and social culture	- Group feeling - Be heard - Competition - Employer
	- Private life/partner	
	- Other	- Rewards
Citizen science elements	- Training	- Knowledge and skills lifestyle and health - Create awareness - Better risk perception - Guidance - More openness to change - Goal setting - Narrative story - Support possibilities in lifestyle change employer
	- Motivational strategies	
	- Social support and culture improvement	- Talk about health with colleagues - Be heard by colleagues and employer - More openness to change
	- Active involvement/citizen scientists	- Group-based - Barriers (Masculine culture, work pressure, work location, negative attitude)
	- More insight in own lifestyle behavior	- Medical screening - Personal diary - Make pictures - Step counter
